# Beyond years: evaluating the impact of surgeon age on outcomes after gastric cancer surgery

**DOI:** 10.1093/bjsopen/zrae016

**Published:** 2024-04-26

**Authors:** Maria Bencivenga, Giuseppe Verlato, Giovanni de Manzoni

**Affiliations:** 1 Department of Surgery, Dentistry, Pediatrics and Gynaecology, Upper GI Surgery Unit, University of Verona, Verona, Italy; Department of Diagnostics and Public Health, Section of Epidemiology and Medical Statistics, University of Verona, Verona, Italy; 1 Department of Surgery, Dentistry, Pediatrics and Gynaecology, Upper GI Surgery Unit, University of Verona, Verona, Italy

We read with interest the manuscript by Leijonmarck *et al.*^1^, examining the role of surgeon age on long-term survival after gastrectomy. This work, based on the linkage of national databases, would be difficult to replicate in our and other countries, due to a strict application of the Global Data Protection Regulation.

The study reflects the management of gastric cancer in Sweden between 2006 and 2015, during which most surgeries were performed open. Surgeon volume of gastrectomy was low: only 25.7% of patients (423 of 1647) were operated on by surgeons performing a mean annual number of ≥6 gastrectomies, with the highest mean volume being 14.75 cases. This low case number likely reflects the low incidence of gastric cancer in Sweden (7.8 per 100 000 in 2020 according to the International Agency for Research on Cancer^2,3^). It might be owing to this limited case number that the adequacy of the surgical procedure was suboptimal in several aspects: lymph node yield was lower than 10 in about 25% of cases, and lower than 18 in about 50% of cases. The 90-day all-cause mortality rate was rather high, amounting to 5.1% (84 of 1647), in accordance with other studies of unselected patients undergoing gastrectomy in European countries^4,5^. Neoadjuvant chemotherapy, which is now widespread, was used in only 31% of cases (511 of 1647).

Analysing the age of the responsible surgeons and grouping this into three groups along tertiles (≤46 years, 47–54 years, ≥55 years), the authors found that ‘Surgeon age ≥47 years might be associated with worse long-term survival in patients who undergo gastrectomy for gastric adenocarcinoma, possibly mediated in part by differences in lymph node yield, resection margin status and complications’. This statement is correct from a statistical point of view. The crude proportions of 5-year all-cause and disease-specific death as well as 90-day all-cause mortality rate increased with increasing surgeon age. The hazard ratio of 5-year disease-specific death in patients operated on by the oldest surgeons with respect to the youngest was 1.40 (95% c.i. 1.18 to 1.65) in the crude univariable model. The hazard ratio decreases to 1.25 (95% c.i. 1.06 to 1.48) but remained significant in a multivariable Cox model adjusting for potential confounders (patient sex, age, education, co-morbidity, pathological tumour stage, tumour sublocation, neoadjuvant therapy). After additional adjustment for lymph node yield, resection margin status, in-hospital complications and annual surgeon volume of gastrectomy, the hazard ratio decreased to 1.17 (95% c.i. 0.98 to 1.39) and was subsequently not significant.

It should be noted that these findings were obtained in an observational retrospective study and so certain biases apply. Older surgeons operated on more difficult cases, with more advanced tumour stages and involving the cardia. Indeed, stages III–IV represented 46.4% of cases operated on by surgeons aged ≥55 years but only 37.4% of cases operated on by surgeons aged <47 years, who conversely managed a larger proportion of stage 0–I cases (30.5% *versus* 23.8%, *P* = 0.008, according to Fisher’s exact test). The cardia was involved in 16.5% of cases operated on by surgeons aged 47–54 years, but in only 8.5% of cases operated on by surgeons aged ≤46 years (*P* < 0.001). Furthermore, patients operated on by older surgeons were less likely to receive neoadjuvant treatment (*P* < 0.001). Adjusting for tumour stage and tumour sublocation can reduce the bias, but not eliminate it. The authors correctly mention an association between surgeon age and long-term outcomes, not a cause–effect relation.

In summary, it remains difficult to interpret the finding that surgeon age <47 years was associated with better long-term outcomes. It would have been highly desirable if the authors had explored and reported the impact of surgeon age on indexes of surgical quality, such as lymph node yield, resection margin status and in-hospital complications in a multivariate analysis, and not only reported crude frequencies. Do younger surgeons perform better surgery than older surgeons when controlling for age and tumour stage in these aspects? Furthermore, some information is missing in the manuscript. For instance, as acknowledged by the authors themselves, the classification of surgeon age (≤46, 47–54, ≥55 years) is not detailed. The authors state that they ‘could not categorize surgeon age into smaller age intervals’, due to power limitations. However, they could have illustrated age distribution in the three age classes by boxplots, so that readers could better appreciate surgeon age, or performed a risk adjusted cumulative sum analysis as in their previous work examining the influence of surgeon age on survival after oesophagectomy^6^.

In our opinion, the main messages of the present study are that, in Sweden, older surgeons tend to take care of more difficult patients, who have lower long-term survival, than younger surgeons do.
